# Predicting Time to Delivery in Hypertensive Disorders: Assessing PlGF and sFlt-1 with the Novel Parameter ‘Mtp-Multiples of a Normal Term Placenta’

**DOI:** 10.3390/jcm13071899

**Published:** 2024-03-25

**Authors:** Valentina Giardini, Alice Angela Francesca Santagati, Elisabetta Marelli, Marco Casati, Anna Cantarutti, Patrizia Vergani

**Affiliations:** 1 Department of Obstetrics, IRCCS San Gerardo dei Tintori Foundation, University of Milano-Bicocca, 20900 Monza, Italy; 2 Department of Obstetrics, MBBM Foundation Onlus, University of Milano-Bicocca, 20900 Monza, Italy; 3 Laboratory Medicine, IRCCS San Gerardo dei Tintori Foundation, 20900 Monza, Italy; marco.casati@irccs-sangerardo.it; 4 Division of Biostatistics, Epidemiology and Public Health, Department of Statistics and Quantitative Methods, University of Milano-Bicocca, 20126 Milano, Italy

**Keywords:** placental growth factor (PlGF), soluble fms-like tyrosine kinase (sFlt-1), hypertensive disorders of pregnancy, placental aging, timing of delivery, angiogenesis, placental dysfunction, duration of pregnancy, retrospective study, singleton pregnancies

## Abstract

**Background:** Imbalanced angiogenesis is characteristic of normal placental maturation but it also signals placental dysfunction, underlying hypertensive disorders during pregnancy. This study aimed to investigate the relationship between angiogenic placental aging, measured by markers placental growth factor (PlGF) and soluble fms-like tyrosine kinase-1 (sFlt-1) using the new index “Multiples of a normal term placenta” (Mtp) and the duration of pregnancy. **Methods:** A retrospective observational study was conducted, including singleton pregnancies diagnosed or suspected of hypertensive disorders after the 20th gestational week. Mtp measures how far a single dosage of angiogenic marker deviates from the expected value in an uncomplicated full-term pregnancy (Mpt = sFlt-1/sFlt-1 reference value or PIGF/PIGF reference value). We considered the 90th, 95th, and 97.5th centiles for sFlt-1 and the 2.5th, 5th, and 10th centiles for PlGF as references. **Results:** The categories with longer time to delivery, regardless of gestational age, were: Mtp PlGF 10th c ≥ 2, ≥3 and Mtp sFlt-1 90th c ≤ 0.5 (median days of 9, 11, 15 days, respectively). These two categories Mtp sFlt-1 90th c ≥ 3 and Mtp sFlt-1 97.5th c ≥ 2 allow the identification of women at risk for imminent delivery within 1 day. Women who were deemed at low/medium risk based on the sFlt-1/PIGF ratio appeared to be at high risk when considering the individual values of sFlt-1 and/or PIGF. **Conclusions:** This new Mtp index for sFlt-1 and PlGF could be employed to assess the degree of placental aging in women with hypertensive disorders. It represents a valid tool for evaluating the risk of imminent birth, irrespective of gestational age, surpassing the current stratification based on the sFlt-1/PIGF ratio.

## 1. Introduction

The placenta is a complex organ that forms during pregnancy, serving multiple functions including gas exchange, metabolic transfer, hormone secretion, and fetal protection. Its development is crucial for fetal growth and the maintenance of a healthy pregnancy [1,2]. The placenta is a transient organ, and therefore its aging is a natural and progressive phenomenon. However, it is also associated with obstetric pathologies in prolonged pregnancies or before post-term, when this process is accelerated [3,4,5].

### 1.1. Placental Aging and Dysfunction

Aging results from the deviation of specific molecular pathways and structures, leading to a deterioration of function at the cellular, tissue, and organ levels. Placental dysfunction is particularly implicated in hypertensive disorders of pregnancy (HDP), such as pre-eclampsia (preE) but also in fetal growth restriction (FGR) [3,6]. Cellular senescence can be induced by various endogenous and exogenous triggers, such as telomere shortening, genomic changes (DNA damage, aneuploidy) and oxidative stress (OS) [7]. OS is a well-known key feature in various placental pathologies, including preE and FGR [8,9]. There is substantial evidence confirming the association between placental aging and placental syndromes, especially preE: (1) Syncytiotrophoblast histopathology in post-term pregnancies is similar to that observed in preE at any gestational age [10]; (2) trophoblast senescence, characterized by telomere shortening, is present in preE and FGR [11]; (3) the placenta from normotensive women who delivered post-term and from women who developed preE exhibits increased the protein expression of senescence markers and oxidative stress, compared to normotensive women who delivered at term [5,12].

### 1.2. Angiogenic Imbalance: Linking Placental Aging and Dysfunction

An imbalanced angiogenesis characterizes normal placental maturation but is also pathognomonic of placental dysfunction [13]. In fact, a correlation has been established between the natural aging process of the placenta and levels of angiogenesis markers in maternal blood, including placental growth factor (PlGF) and soluble fms-like tyrosine kinase-1 (sFlt-1). PlGF is an angiogenic protein secreted by the syncytiotrophoblast, promoting placental angiogenesis and vascular homeostasis [14]. On the other hand, sFlt-1 is a circulating anti-angiogenic protein that adheres to the receptor-binding domains of PlGF and vascular endothelial growth factor (VEGF), preventing their interaction with endothelial receptors and inducing endothelial dysfunction. sFlt1 is released from the trophoblast in response to various stressors such as hypoxia and oxidative stress [15,16]. In a healthy pregnancy, the maternal serum levels of PlGF rise steadily up to 29–32 weeks and then decrease until delivery, while sFlt-1 concentrations increase towards the term of pregnancy [8]. Women affected by preE show reduced serum levels of PlGF and elevated levels of sFlt-1 before clinical symptoms appear. [17]. Currently, the sFlt1/PlGF ratio is used as a clinical biomarker for the early detection and prognosis of preE [18]. Recently, it has also been suggested that low PlGF concentrations are associated with FGR [19]. Therefore, these two markers, initially studied as predictors of preE, have emerged as predictors of placental dysfunction and syncytiotrophoblast aging. They reveal that the duration of pregnancies depends on placental capacity, such that there is increasing placental dysfunction at and beyond term [17].

### 1.3. Angiogenic Markers as Predictors of Pregnancy Duration

An association between angiogenic markers and pregnancy duration has been demonstrated in many studies: the PELICAN study showed that low PlGF (i.e., <100 pg/mL) before 35 weeks’ gestation had high sensitivity and high negative predictive value in the diagnosis of preE requiring delivery within 14 days [20]. The PETRA study demonstrated that the median time to delivery was 10 days in women with suspected preE before 35 weeks and low PlGF (i.e., 12–100 pg/mL) and 2 days for very low PlGF (i.e., <12 pg/mL) [21]. Rana and colleagues found that delivery occurred within 2 weeks in 86.0% of women with an sFlt-1/PlGF ratio > 85 before 34 weeks’ gestation [22]. Verlohren et al. reported that a very high sFlt-1/PlGF ratio (i.e., >655 before the 34th week of gestation and >201 at or after the 34th week of gestation) is often associated with the need to deliver within 48 h [23].

In light of these considerations, our study aimed to investigate the potential of the new index “Multiples of a normal term placenta” (Mtp) in predicting the duration of pregnancy by examining its association with placental angiogenic aging. Specifically, we analyzed the median time to delivery in hospitalized pregnant women with a diagnosis or suspicion of hypertensive disorder, comparing their angiogenic markers with those of uncomplicated full-term pregnancies using the Mtp index.

## 2. Materials and Methods

A retrospective observational study was conducted to investigate the relationship between angiogenic markers and the timing of birth in women with HDP. The study included 182 women hospitalized at the Obstetrics Unit of San Gerardo Hospital in Monza, Italy, between May 2018 and December 2020. All women had received a diagnosis or had a suspicion of HDP after 20 weeks of gestation and had undergone at least one measurement of the PlGF and sFlt-1 markers.

All patients provided informed consent for the collection of biological samples. The primary objective was to predict the timing of delivery in these complicated hypertensive pregnancies based on the angiogenic profile, considering uncomplicated full-term pregnancies as a reference. The angiogenic profile was evaluated using the ‘Multiples of a normal term placenta’ (Mtp) index. The secondary outcome compared the Mtp index with the currently referenced sFlt-1/PlGF ratio, derived from Verlohren’s studies [24].

Mtp is a measure of how far a single dosage of an angiogenic marker deviates from the value expected in an uncomplicated full-term (≥37 weeks) pregnancy. The reference values include the 90th (7901 pg/mL), 95th (9184 pg/mL), and 97.5th (11,471 pg/mL) for sFlt-1, and the 2.5th (48.9 pg/mL), 5th (54.4 pg/mL), and 10th (68.6 pg/mL) centile (c) for PlGF at term (Table 1). These values were obtained from the Prospective Multicenter Study: Diagnosis of Preeclampsia by means of the Elecsys sFlt-1 assay and the Elecsys PlGF assay (Roche study no. CIM RD000556/X06P006)—Table 1 [25]):

$$Mtp\;sFlt-1\;y\;c=\frac{marker\;serum\;value\;of\;sFlt-1}{90th,95th,97.5th\;centile\;of\;sFlt-1*}\;Mtp\;PIGF\;z\;c=\frac{marker\;serum\;value\;of\;PIGF}{2.5th,5th,10th\;centile\;of\;PIGF*}$$

y = 90th or 95th or 97.5th centile;

z = 2.5th or 5th or 10th centile.

∗ Values obtained from full-term pregnancies with normal outcomes.

**Table 1:** Serum values of the sFlt-1 (pg/mL) and PlGF (pg/mL) in full term pregnancy with normal outcome used as a reference for the calculation of Mtp [26].

Centiles	≥37 Weeks
sFlt-1	
90	7901
95	9184
97.5	11,471
PlGF	
2.5	48.9
5	54.4
10	68.6

### 2.1. Link for Calculating Mtps Using Excel: Supplementary Materials

For example, a patient’s Mtps at 34 weeks and 4 days of pregnancy with a PlGF level of 24.48 pg/mL and an sFlt-1 level of 17,794 pg/mL–sFlt-1/PIGF ratio = 726.88, were calculated as follows:

Mtp PlGF 2.5th c = 24.48/48.9 = 0.5

Mtp PlGF 5th c = 24.48/54.4 = 0.5

Mtp PlGF 10th c = 24.48/68.6 = 0.4

Mtp sFlt-1 90th c = 17,794/7901 = 2.3

Mtp sFlt-1 95th c = 17,794/9184 = 1.9

Mtp sFlt-1 97.5th c = 17,794/11,471 = 1.6

This preeclamptic patient’s pregnancy ended 2 days after dosing, and her serum PlGF levels were about half of those in an uncomplicated full-term pregnancy, while her serum sFlt-1 levels were approximately double when considering the 90–95th centile as a reference for sFlt-1.

sFlt-1 and PlGF were measured in serum samples on the Cobas e601 platform (Roche Diagnostics, Indianapolis, IN, USA) using the electrochemiluminescence immunoassay principle (REF 05109523190 and 05144671190, respectively). According to EP05-A3 CLSI protocol, the reproducibility of serum PlGF and serum sFlt-1 at different concentration levels (6.8–9219 pg/mL and 53.2–70,552 pg/mL, respectively) was less than 5%.

The study included 240 dosages of PlGF and sFlt-1, 153 (64%) before 37 weeks of gestation, and 81 (34%) before the 34th week.

The sFlt-1/PlGF ratio was classified into four categories based on the following ratios: low (<38), medium (38–85/110 after the 34th gestational week), high (>85 or >110 after the 34th gestational week), and very high-risk (>655 or >201 after the 34th gestational week). A sFlt-1/PlGF ratio greater than 85 (for gestational weeks 20 to 33 and 6 days) and greater than 110 (from 34 weeks to delivery) has been shown to be highly suggestive of preE [26]. The PROGNOSIS study data demonstrated the ability of the sFlt 1/PlGF ratio cut-off of 38 to predict a combined endpoint of preE, eclampsia, or HELLP syndrome or maternal or fetal negative outcomes [27].

Medical management, including hospitalization, expectant management, and delivery, was determined according to our protocol based on national and international guidelines. Therefore, the defining time point of delivery was independent of the values of biochemical markers but rather based on other maternal–fetal clinical and laboratory parameters.

### 2.2. Statistical Analysis

The study was descriptive in nature. The median time to delivery was calculated in the following categories, overall and subdivided by gestational age <34 and <37 weeks of gestation, considering all available dosages carried out in these patients from the time of admission to delivery:

sFlt-1/PlGF low–medium–high–very high,

Mtp PlGF 10th c ≤ 0.5, <1, ≥ 2 and ≥3,

Mtp PlGF 5th c ≤ 0.5 and ≤1,

Mtp PlGF 2.5th c ≤ 0.5 and ≤1,

Mtp sFlt-1 90th c ≤ 0.5, >1, ≥2, and ≥3,

Mtp sFlt-1 95th c ≥ 1 and ≥2,

Mtp sFlt-1 97.5th c ≥ 1 and ≥2,

Mtp PlGF 10th c < 1 and Mtp sFlt-1 90th c > 1,

Mtp PlGF 5th c ≤ 1 and Mtp sFlt-1 95th c ≥ 1,

Mtp PlGF 2.5th c ≤ 1 and sFlt-1 97.5th c ≥ 1.

Discrete variables were reported as numbers and percentages, and continuous variables were reported as median and interquartile range (IQR).

## 3. Results

Table 2 shows the median and interquartile range (IQR) of Mtp, considering as a reference 2.5th c, 5th c, and 10th c for PlGF and 90th c, 95th c, and 97.5th c for sFlt-1, in the four classes (low, medium, high, and very high) of sFlt-1/PlGF ratio with respect to the 34th gestational week. In the low sFlt-1/PlGF ratio category, the maternal serum levels of PlGF are significantly elevated, reaching a median Mtp of 4.4 (IQR 3.2 to 9.3), approximately four times higher than that observed in a full-term uncomplicated pregnancy when considering the 2.5th centile reference value for PlGF. Meanwhile, sFlt-1 levels are markedly reduced, with a median Mtp of 0.4 (IQR 0.3 to 0.6), considering the 90th centile of sFlt-1. On the contrary, in the very high category, PlGF levels are halved (median Mtp 0.5, IQR 0.2 to 0.8) compared to the 10th c, while sFlt-1 concentrations are elevated (median Mtp 1.7, IQR 1.2 to 2.1), nearly double with respect to the 90th c.

The data on median time to delivery (days) in the four classes of sFlt-1/PlGF ratio are reported in Table 3: as the sFlt-1/PlGF ratio increases, the time to delivery decreases, and this trend is particularly pronounced when measurements are taken before 37 weeks of gestation. In the <37 weeks group, patients with low/medium/high/very high ratios delivered after 17, 10, 7, and 4 days, respectively. For an even earlier gestational age <34 weeks, the days to delivery increased in the case of a low ratio (49 days), remained similar for medium–high ratios (10 and 7 days, respectively), and slightly decreased in the very high category (2 days).

Table 4 shows the median time to delivery in the various categories of Mtp considering all dosages, those before the 37th gestational week and the 34th week.

The categories with longer time to delivery, regardless of gestational age, were: Mtp PlGF 10th c ≥ 2, ≥ 3 and Mtp sFlt-1 90th c ≤ 0.5. The median number of days between the dosage and delivery in all patients was 9, 11, and 15 days, respectively, and were greater before the 37th week (15, 22, and 24 days). The categories with shorter time to delivery, within 7 days, regardless of the gestational age, in descending order, were: Mtp PlGF 10th c ≤ 0.5, Mtp PlGF 10th c < 1 and Mtp sFlt-1 90th c > 1, Mtp PlGF 10th c < 1, Mtp PlGF 5th c ≤ 1, Mtp PlGF 2.5th c ≤ 1, Mtp PlGF 5th c ≤ 1 and Mtp sFlt-1 95th c ≥ 1, Mtp PlGF 5th c ≤ 0.5, Mtp PlGF 2.5th c ≤ 0.5, Mtp sFlt-1 90th c > 1, Mtp sFlt-1 90th c ≥ 2, Mtp sFlt-1 95th c ≥ 1, Mtp sFlt-1 95th c ≥ 2, Mtp sFlt-1 97.5th c ≥ 1, Mtp PlGF 2.5th c ≤ 1 and Mtp sFlt-1 97.5th c ≥ 1, Mtp sFlt-1 90th c ≥ 3, Mtp sFlt-1 97.5th c ≥ 2. It is of note that the last two categories allow the identification of women at risk for imminent delivery within 1 day.

In Table 5, we reported the stratification of the Mtp categories according to the four classes of sFlt-1/PlGF. All patients with a Mtp PlGF 10th c ≥ 3 had a low sFlt-1/PlGF ratio. The Mtp classes associated with shorter time to delivery were distributed not only in the very high ratio category. For example, 57% (8/14) of patients belonging to the two categories Mtp sFlt-1 90th c ≥ 3 and Mtp sFlt-1 97.5th c ≥ 2 with risk of imminent delivery were considered only high risk according to Verlohren’s categories.

## 4. Discussion

Our data confirm a correlation between placental angiogenesis and the timing of delivery. Placental angiogenesis is a key process in the development and function of the placenta, the main source of circulating angiogenic factors during pregnancy; for this reason, these biomarkers, including PlGF and sFlt-1, indicate that the duration of pregnancy is constrained by placental function (8), and an angiogenic imbalance is a characteristic of both placental aging and certain placental pathologies [17,28].

Nowadays, angiogenic assessment during pregnancy is a crucial component of obstetric care, facilitated by fully automated, commercially available assays. A growing body of evidence suggests that PlGF-based testing improves the diagnosis of placental dysfunction, reducing adverse fetal–maternal outcomes through appropriate risk stratification and resource redistribution [16,24].

A correlation has been proven between the natural process of the aging of the placenta and levels of angiogenesis markers in maternal blood: in healthy pregnancies close to term, there is a gradual decrease in PlGF and an increase in sFlt-1, resembling the pattern observed in women with preE [17].

Currently, the sFlt-1/PlGF ratio appears to have better diagnostic performance for preE than individual biomarkers. Moreover, it is also utilized to identify women at risk of imminent delivery [23].

### 4.1. The Significance of the Mtp Index in Hypertensive Disorders of Pregnancy: Insights into Placental Aging and Angiogenic Profile

The important finding of our study is the new Mtp index for PlGF and sFlt-1 (alone or in combination) as a unit of measurement of pregnancy duration in hypertensive disorders, regardless of gestational age. We tested the hypothesis that the angiogenic profile of the placenta of uncomplicated full-term pregnancies can be considered the reference for defining a condition at risk of imminent birth.

There is substantial evidence confirming the association between placental aging and hypertensive disorders of pregnancy, particularly preE, both histologically and biochemically. In a normal pregnancy, as the natural aging process of the placenta begins, hypoperfusion foci develop, with their numbers increasing as gestational age advances. The elevation in the number of hypoperfusion foci correlates with the levels of markers indicating disordered angiogenesis (sFlt-1/PlGF) [29].

Premature placental aging is the consequence of OS-induced damage to lipids, proteins and DNA in placental tissue that may cause cellular senescence or cell death in the placenta, leading to placental dysfunction and insufficiency [7]. This concept is clearly shown in Table 2. It shows the median of Mtp for PlGF and sFlt-1 in the four classes of sFlt-1/PlGF ratio: the angiogenic profile gradually ages from a low to a very high ratio with a decrease in PlGF and the release of sFIt-1, causing generalized endothelial dysfunction in maternal circulation. In the low sFlt-1/PlGF ratio category, maternal serum levels of PlGF are significantly elevated, reaching an Mtp = 4.4, approximately four times higher than that observed in a full-term uncomplicated pregnancy when considering the 2.5th centile reference value for PlGF. Meanwhile, sFlt-1 levels are markedly reduced, with an Mtp = 0.4, considering the 90th centile of sFlt-1. On the contrary, in the very high category, PlGF levels are halved (Mtp = 0.5) compared to the 10th c, while sFlt-1 concentrations are elevated (Mtp = 1.7). Scaife et al. demonstrated that placental oxidative stress and senescence increase in parallel with a reduction in PlGF expression as normotensive pregnancy progresses, while antioxidant defenses diminish as gestational age increases. These features become evident earlier in gestation in women with preE, suggesting accelerated senescence, probably secondary to their poor antioxidant status [5]. In clinically healthy post-term pregnancies, Bowe et al. showed that an antiangiogenic predelivery profile (lower PlGF level and higher sFlt-1/PlGF ratio) was associated with a composite adverse delivery outcome likely due to a placental cause [30].

### 4.2. Insights into Pregnancy Duration from Angiogenic Marker Analysis: Past Studies and the Role of Mtp

An association between angiogenic markers and pregnancy duration has already been highlighted in the literature, but almost always before the 37th week [20,21,22,23]. Our analysis considered all gestational periods, stratifying for gestational age <37 and <34 weeks. This approach was chosen to account for the fact that it is easier to decide to terminate a pregnancy at its end, reducing potential bias. Verlohren et al. reported that a significantly elevated sFlt-1/PlGF ratio (>655 before the 34th week of gestation and >201 at or after the 34th week of gestation) is often linked to the need for delivery within 48 h [23]. Our findings also confirmed an inverse correlation between the sFlt-1/PlGF ratio and time to delivery. With the introduction of the new parameter “Mtp” for PlGF and sFlt-1, whether used alone or in combination, we were able to further stratify Verlohren’s categories, particularly the high-risk category. This allowed us to avoid underestimating high-risk cases with a low/medium ratio, as demonstrated in Table 5.

The categories associated with longer time to delivery, irrespective of gestational age, included sFlt-1/PlGF low, Mtp PlGF 10° c ≥ 2 and ≥3, and Mtp sFlt-1 90th c ≤ 0.5. On the other hand, those associated with a shorter time to delivery (within 1 day) comprised Mtp sFlt-1 90th c ≥ 3 and Mtp sFlt-1 97.5th c ≥ 2.

We posit that a threshold limit of tolerance exists for both the mother and the fetus concerning these angiogenic factors: adequate levels of PlGF are crucial to ensure the growth and well-being of the fetus, while excessive levels of sFlt-1 can be harmful to the maternal endothelium [15,16]. Our group further substantiated this hypothesis by demonstrating that excessive sFlt-1 levels, indicated by a cut-off value of sFlt-1 ≥ 15,802 pg/mL (equivalent to Mtp sFlt-1 90th c ≥ 2), are associated with serious obstetric complications in multiple pregnancies hospitalized for an HDP/FGR disorder, regardless of gestational age and chorionicity [31].

Therefore, analyzing the two components, PlGF and sFlt-1, individually rather than solely focusing on the ratio enables us to better assess the risk of imminent birth and determine the type of surveillance required, including the need for respiratory distress syndrome (RDS) prophylaxis.

### 4.3. Study Limitations and Future Directions

Our study is limited by a small sample size, and the dosage of angiogenic markers may vary depending on the method used; different methods and analyzers might yield different results.

Prospective data with larger sample sizes and standardization across assay methods are necessary to determine which cut-offs of PlGF and sFlt-1 are indicative of maternal–fetal complications that would warrant the termination of pregnancy.

Additionally, it is prudent to approach the interpretation of marker levels cautiously in the presence of pharmacological therapies, uncontrolled diabetes mellitus/gestational diabetes, and infections, as these factors may contribute to fluctuations in placental angiogenesis that are yet to be fully understood [24,32,33].

## 5. Conclusions

In conclusion, our study supports the clinical integration of angiogenic biomarkers in women with hypertensive disorders to identify patients at risk of imminent delivery, even beyond the 37th week.

We propose a novel interpretation of angiogenic markers: the Mtp index for PlGF and sFlt-1 can be employed to assess the degree of placental aging and serves as a valuable tool for evaluating the risk of imminent birth irrespective of gestational age, outperforming the current stratification methods in use. Therefore, it is essential to educate obstetricians on interpreting these markers in clinical practice as integrative tools for surveillance, though not mandatory, in determining the timing or mode of delivery. Test results should always be contextualized to minimize maternal–fetal complications as well as iatrogenic delivery. Future studies on angiogenic markers are warranted.

## Figures and Tables

**Table 2:** Median and interquartile range (IQR) of the multiples of a normal term placenta (Mtp) in the four classes of sFlt-1/PlGF ratio with respect to the 34th gestational week (low <38, medium 38–85/110, high >85 or >110, and very high >655 or >201) considering as references the 2.5th, 5th, and 10th centile (c) for PlGF and 90th, 95th, and 97.5th c for sFlt-1 [Median [IQR]; *n* (%)].

	sFlt-1/PlGF			
	*n* = 240			
Mtp	Low	Medium	High	Very High
	56 (23)	71 (30)	80 (33)	33 (14)
PlGF 2.5th c	4.4 [3.2–9.3]	2.0 [1.6–2.4]	1.2 [0.7–1.7]	0.7 [0.3–1.1]
PlGF 5th c	3.9 [2.8–8.3]	1.8 [1.5–2.2]	1.1 [0.6–1.6]	0.6 [0.3–1.0]
PlGF 10th c	3.1 [2.3–6.6]	1.4 [1.2–1.7]	0.9 [0.5–1.2]	0.5 [0.2–0.8]
sFlt-1 90th c	0.4 [0.3–0.6]	0.9 [0.7–1.1]	1.4 [1.0–1.8]	1.7 [1.2–2.1]
sFlt-1 95th c	0.3 [0.2–0.5]	0.7 [0.6–0.9]	1.2 [0.9–1.6]	1.4 [1.0–1.8]
sFlt-1 97.5th c	0.3 [0.2–0.4]	0.6 [0.5–0.7]	0.9 [0.7–1.3]	1.2 [0.8–1.5]

**Table 3:** Median and interquartile range (IQR) time to delivery (days) in the four classes of sFlt-1/PlGF ratio with respect to the 34th gestational week (low <38, medium 38–85/110, high >85, or >110 and very high >655 or >201), considering all dosages, those before the 37th gestational week and the 34th week [Median [IQR]; *n* (%)].

sFlt-1/PlGF	Time to Delivery					
	All		*n* < 37 Weeks		*n* < 34 Weeks	
	*n* = 240		*n* = 153 (64)		*n* = 81 (34)	
	*n*	Days	*n*	Days	*n*	Days
low	56 (23)	10 [4–23]	40 (26)	17 [9–28]	15 (18)	49 [25–65]
medium	71 (30)	4 [2–9]	25 (16)	10 [5–16]	7 (9)	10 [4–34]
high	80 (33)	6 [2–12]	63 (42)	7 [3–14]	48 (59)	7 [3–14]
very high	33 (14)	3 [1–6]	25 (16)	4 [2–6]	11(14)	2 [0–5]

**Table 4:** Median and interquartile range (IQR) of the time to delivery (days) in the various categories of Mtp considering all dosages, those before the 37th gestational week and the 34th week [median [IQR]; *n* (%)].

Mtp Category	Time to Delivery					
	All		*n* < 37 Weeks		*n* < 34 Weeks	
	*n* = 240		*n* = 153 (64)		*n* = 81 (34)	
	*n*	Days	*n*	Days	*n*	Days
Mtp PlGF 10th c ≤ 0.5	47 (20)	6 [2–10]	42 (28)	6 [2–12]	35 (43)	6 [2–12]
Mtp PlGF 10th c < 1	89 (37)	5 [2–10]	71 (46)	6 [2–13]	50 (62)	6 [3–14]
Mtp PlGF 10th c ≥ 2	60 (25)	9 [4–22]	40 (26)	15 [7–27]	17 (21)	27 [23–61]
Mtp PlGF 10th c ≥ 3	29 (12)	11 [4–27]	21 (14)	22 [8–51]	11 (14)	51 [25–70]
Mtp PlGF 5th c ≤ 0.5	30 (13)	4 [1–7]	28 (18)	5 [2–8]	27 (33)	6 [2–9]
Mtp PlGF 5th c ≤ 1	69 (29)	5 [2–10]	56 (37)	6 [3–13]	41 (51)	6 [3–14]
Mtp PlGF 2.5th c ≤ 0.5	21 (9)	4 [1–6]	18 (12)	4 [1–7]	17 (21)	4 [2–7]
Mtp PlGF 2.5th c ≤ 1	64 (27)	5 [2–10]	54 (35)	6 [2–12]	41 (51)	6 [3–14]
Mtp sFlt-1 90th c ≤ 0.5	55 (23)	15 [4–28]	37 (24)	24 [15–43]	20 (25)	41 [26–55]
Mtp sFlt-1 90th c > 1	109 (45)	4 [2–7]	76 (50)	6 [2–10]	45 (56)	5 [1–10]
Mtp sFlt-1 90th c ≥ 2	24 (10)	3 [1–7]	19 (12)	4 [2–9]	13 (16)	3 [0–14]
Mtp sFlt-1 90th c ≥ 3	7 (3)	1 [0–2]	5 (3)	0 [0–2]	4 (5)	0 [0–1]
Mtp sFlt-1 95th c ≥ 1	98 (41)	3 [2–7]	71 (46)	6 [2–9]	42 (52)	5 [1–8]
Mtp sFlt-1 95th c ≥ 2	16 (7)	3 [1–6]	12 (8)	4 [2–9]	9 (11)	3 [0–14]
Mtp sFlt-1 97.5th c ≥ 1	63 (26)	3 [1–7]	45 (29)	4 [2–7]	29 (36)	3 [1–7]
Mtp sFlt-1 97.5th c ≥ 2	7 (3)	1 [0–2]	5 (3)	0 [0–2]	4 (5)	0 [0–1]
Mtp PlGF 10th c < 1 and Mtp sFlt-1 90th c > 1	57 (24)	6 [2–8]	51 (33)	6 [2–9]	35 (43)	6 [2–14]
Mtp PlGF 5th c ≤ 1 and Mtp sFlt-1 95th c ≥ 1	40 (17)	5 [2–7]	35 (23)	6 [2–8]	25 (31)	6 [2–7]
Mtp PlGF 2.5th c ≤ 1 and Mtp sFlt-1 97.5th c ≥ 1	22 (9)	3 [1–7]	20 (13)	4 [2–7]	17 (21)	6 [2–7]

**Table 5:** Stratification of the various categories of Mtp according to the four classes of sFlt-1/PlGF ratio with respect to the 34th gestational week (low <38, medium 38–85/110, high >85 or >110, and very high >655 or >201) [*n* (%)].

Mtp Category	*n*	sFlt-1/PlGF			
		Low	Medium	High	Very High
Mtp PlGF 10th c ≤ 0.5	47	0	2 (4)	26 (56)	19 (40)
Mtp PlGF 10th c < 1	89	0	11 (12)	48 (54)	30 (34)
Mtp PlGF 10th c ≥ 2	60	47 (78)	9 (15)	4 (7)	0
Mtp PlGF 10th c ≥ 3	29	29 (100)	0	0	0
Mtp PlGF 5th c ≤ 0.5	30	0	0	15 (50)	15 (50)
Mtp PlGF 5th c ≤ 1	69	0	7 (10)	35 (51)	27 (39)
Mtp PlGF 2.5th c ≤ 0.5	21	0	0	7 (33)	14 (67)
Mtp PlGF 2.5th c ≤ 1	64	0	5 (8)	35 (55)	24 (37)
Mtp sFlt-1 90th c ≤ 0.5	55	40 (73)	12 (22)	3 (5)	0
Mtp sFlt-1 90th c > 1	109	2 (2)	18 (17)	57 (52)	32 (29)
Mtp sFlt-1 90th c ≥ 2	24	0	0	14 (58)	10 (42)
Mtp sFlt-1 90th c ≥ 3	7	0	0	4 (57)	3 (43)
Mtp sFlt-1 95th c ≥ 1	98	1 (1)	14 (14)	53 (54)	30 (31)
Mtp sFlt-1 95th c ≥ 2	16	0	0	10 (63)	6 (37)
Mtp sFlt-1 97.5th c ≥ 1	63	1 (1)	3 (5)	39 (62)	20 (32)
Mtp sFlt-1 97.5th c ≥ 2	7	0	0	4 (57)	3 (43)
Mtp PlGF 10th c < 1 and Mtp sFlt-1 90th c > 1	57	0	0	28 (49)	29 (51)
Mtp PlGF 5th c ≤ 1 and Mtp sFlt-1 95th c ≥ 1	40	0	0	16 (40)	24 (60)
Mtp PlGF 2.5th c ≤ 1 and Mtp sFlt-1 97.5th c ≥ 1	22	0	0	11 (50)	11 (50)

## Data Availability

The original contributions presented in the study are included in the article/Supplementary Materials, further inquiries can be directed to the corresponding authors.

## Supplementary Materials

The following supporting information can be downloaded at: https://www.mdpi.com/article/10.3390/jcm13071899/s1. File S1: Mtp calculator.xlsx.
